# Factors Associated With the Spatial Distribution of Severe Fever With Thrombocytopenia Syndrome in Zhejiang Province, China: Risk Analysis Based on Maximum Entropy

**DOI:** 10.2196/46070

**Published:** 2024-08-02

**Authors:** Mingyong Tao, Ying Liu, Feng Ling, Jiangping Ren, Rong Zhang, Xuguang Shi, Song Guo, Jianmin Jiang, Jimin Sun

**Affiliations:** 1 Hangzhou Center for Disease Control and Prevention, Hangzhou, China; 2 Key Laboratory of Vaccine, Prevention and Control of Infectious Disease of Zhejiang Province, Zhejiang Provincial Center for Disease Control and Prevention, Hangzhou, China

**Keywords:** severe fever with thrombocytopenia syndrome, MaxEnt, maximum entropy, tick density, spatial distribution, risk factor, China

## Abstract

**Background:**

Severe fever with thrombocytopenia syndrome (SFTS) is an emerging infectious disease that was first identified in mainland China in 2009 and has been reported in Zhejiang Province, China, since 2011. However, few studies have focused on the association between ticks, host animals, and SFTS.

**Objective:**

In this study, we analyzed the influence of meteorological and environmental factors as well as the influence of ticks and host animals on SFTS. This can serve as a foundational basis for the development of strategic policies aimed at the prevention and control of SFTS.

**Methods:**

Data on SFTS incidence, tick density, cattle density, and meteorological and environmental factors were collected and analyzed using a maximum entropy–based model.

**Results:**

As of December 2019, 463 laboratory-confirmed SFTS cases were reported in Zhejiang Province. We found that the density of ticks, precipitation in the wettest month, average temperature, elevation, and the normalized difference vegetation index were significantly associated with SFTS spatial distribution. The niche model fitted accurately with good performance in predicting the potential risk areas of SFTS (the average test area under the receiver operating characteristic curve for the replicate runs was 0.803 and the SD was 0.013). The risk of SFTS occurrence increased with an increase in tick density, and the response curve indicated that the risk was greater than 0.5 when tick density exceeded 1.4. The risk of SFTS occurrence decreased with increased precipitation in the wettest month, and the risk was less than 0.5 when precipitation exceeded 224.4 mm. The relationship between elevation and SFTS occurrence showed a reverse V shape, and the risk peaked at approximately 400 m.

**Conclusions:**

Tick density, precipitation, and elevation were dominant influencing factors for SFTS, and comprehensive intervention measures should be adjusted according to these factors to reduce SFTS incidence in Zhejiang Province.

## Introduction

Severe fever with thrombocytopenia syndrome (SFTS) is an emerging infectious disease that was initially identified in 2009 and subsequently reported in 2011 [1]. The most common clinical symptoms of SFTS cases include fever, thrombocytopenia, leukocytopenia, gastrointestinal symptom, gingival hemorrhage, conjunctival congestion, and central nervous system manifestations. Although most patients with SFTS have self-limited clinical manifestations, some patients also develop severe cases and die due to multiple organ failure [1-3]. The case fatality rate of SFTS was initially reported to be up to 30%, but it has shown a downward trend in recent years [4]. Of note, the annual number of SFTS cases had an increasing trend, and the affected areas expanded. It was endemic in mainland China, South Korea, and Japan, and the severe fever with thrombocytopenia syndrome virus (SFTSV) was also detected in the United Arab Emirates, Vietnam, and Myanmar [5-8].

Previous studies reported that SFTSV can be transmitted through direct contact with secretions or blood of patients with SFTS and that there is probable aerosol transmission, while the majority of SFTS cases were infected through tick bites [3,9,10]. SFTSV has been detected in *Haemaphysalis longicornis*, *Rhipicephalus microplus*, *Amblyomma testudinarium*, and *Ixodes nipponensis,* and *H. longicornis* is considered to be most important for the epidemiologic maintenance and transmission of SFTSV [11,12]. Luo et al [13] reported that *H. longicornis* ticks that fed on SFTSV-infected mice could acquire the virus and transstadially and transovarially transmit it to other ticks in different developmental stages, and SFTSV-infected ticks could transmit the virus to mice during feeding. SFTSV was also found in domestic and wild animals, such as goats, sheep, cattle, dogs, pigs, and chickens [14].

Zhejiang Province is one of the southeastern coastal provinces of mainland China, which has a Cfa climate, specifically indicating a “humid subtropical climate” according to the Köppen climate classification system. This climate is characterized by warm temperatures, high humidity, and hot summers. More than 400 SFTS cases have been reported in Zhejiang Province since 2011 [15,16].

Liu et al [17] analyzed the spatial distribution of SFTS in Xinyang City, Henan Province, using Poisson regression analysis based on the SFTS epidemic data from 2011 to 2012 and concluded that the spatial distribution of SFTS was significantly correlated with the coverage ratio of shrubs, forests, and farmlands. Du et al [18] constructed an ecological niche model (ENM) based on the epidemic data from January 2010 to April 2013 in Shandong Province, combined with biological factors and meteorological data, suggesting that the key environmental factors affecting the occurrence of SFTS are temperature, precipitation, land cover, the normalized difference vegetation index (NDVI), and duration of sunlight. Sun et al [19] constructed an ENM based on the epidemic data from 2011 to 2018 in mainland China and found that when the annual average temperature is between 12.5 and 17.5 °C, the annual cumulative precipitation is between 700 and 2250 mm, and the annual relative humidity is between 63% and 82%, the likelihood of SFTS occurrence is very high. In Miyazaki Prefecture, Japan, geographically weighted regression was conducted on a scale of 10×10 km using a Geographic Information System, and it was found that altitude and the proportion of farmland area in the geographical grid are factors affecting SFTS [20]. However, few studies focused on the association between ticks, host animals, and SFTS.

The objective of this study is to conduct an analysis on the impact of meteorological and environmental factors, as well as ticks and host animals, on the incidence of SFTS. Furthermore, it aims to investigate how these factors specifically contribute to the occurrence of the disease and develop a comprehensive prediction model. The insights garnered from this study are anticipated to underpin the formulation of evidence-based, strategic policies that are pivotal in the realm of SFTS prevention and control.

## Methods

### Study Area

**Figure 1.**
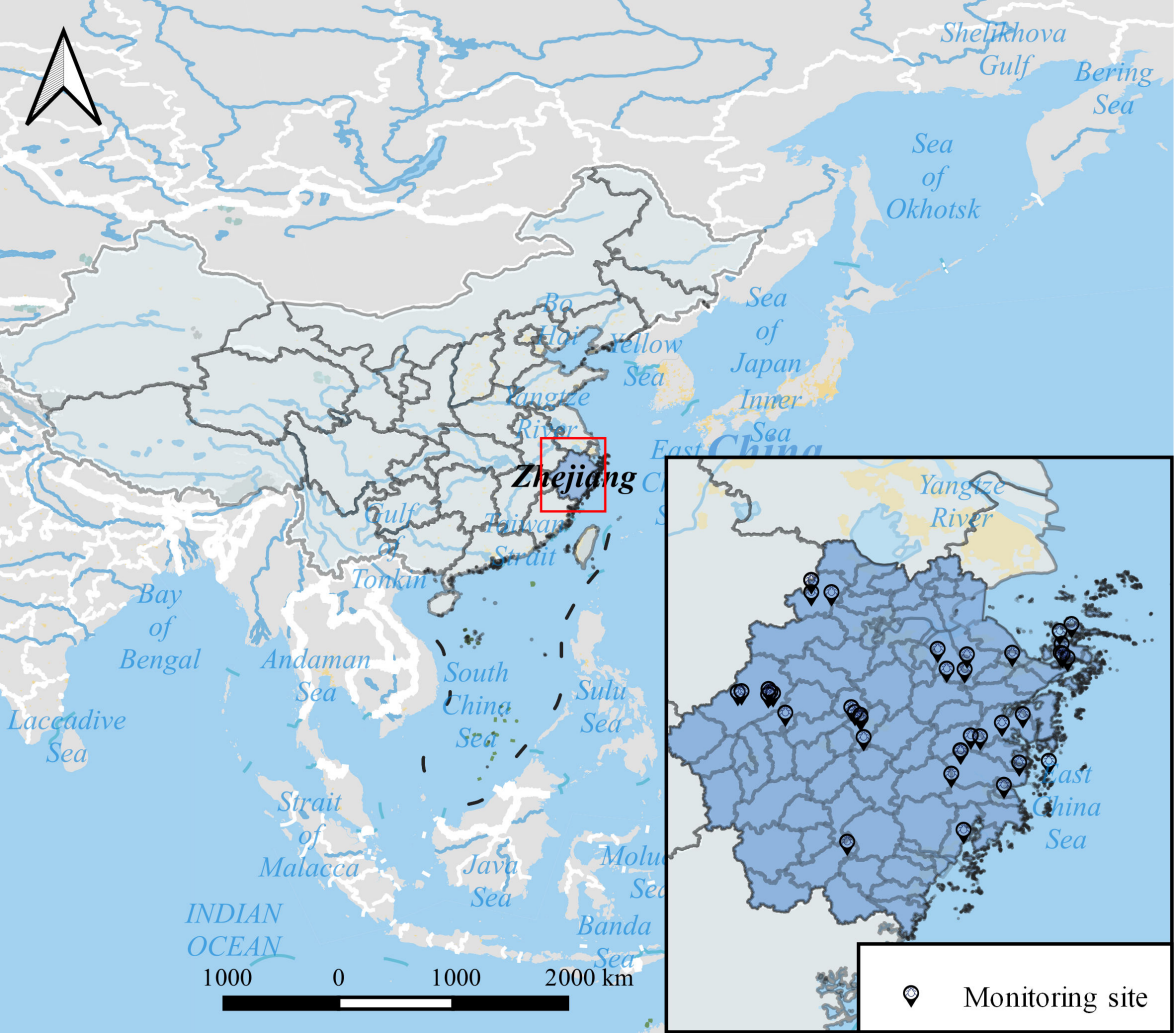
The location of Zhejiang Province and tick monitoring sites.

Zhejiang Province is one of the southeastern coastal provinces of China, located 27°02’N to 31°11’N and 118°01’E to 123°10’E. Zhejiang Province is in the southern wing of the Yangtze River Delta, adjacent to Shanghai City and Jiangsu Province in the north, Anhui Province and Jiangxi Province in the west, and Fujian Province in the south (Figure 1). According to the climate Köppen-Geiger classification, Zhejiang Province has a Cfa climate characterized by a warm temperature, full humidity, and a hot summer. The ecosystem types in Zhejiang Province mainly include forest, wetland, ocean, farmland, city, and grassland, among others [21]. Zhejiang Province, as indicated by its high NDVI, is abundant in forest resources. These forests are predominantly found in the subtropical evergreen broad-leaved forest region, specifically within the humid evergreen broad-leaved forest area of the subtropical zone. The province boasts a forest coverage rate of 61.2%, with a total forest area of 6,609,500 hectares, more than 60% of which consists of natural forests. [22].

### Data Collection

Suspected cases of SFTS are defined by individuals with any of the following epidemiological backgrounds and consistent clinical manifestations: (1) a history of working, living, or traveling in hilly, forested, or mountainous areas during the epidemic season; (2) a history of tick bite within 2 weeks prior to the onset of illness; or (3) a history of contact with infected animals or confirmed cases of SFTS. Clinically diagnosed cases are suspected cases that meet any of the following criteria: (1) a positive test for SFTSV-specific immunoglobulin M antibodies and (2) evidence of multiorgan dysfunction. Confirmed cases are either suspected or clinically diagnosed cases that fulfill any of the following: (1) a positive SFTSV nucleic acid test, (2) isolation of SFTSV from clinical specimens, and (3) seroconversion for SFTSV-specific immunoglobulin G antibodies or a 4-fold or greater increase in antibody titer from the acute phase to the recovery phase. The data of SFTS cases including the date of illness onset and residential address were obtained from the China Information System for Diseases Control and Prevention.

Meteorological data including the hours of sunshine (DEMO1), average relative humidity (DEMO2), average land surface temperature (DEMO3), 20‐8 precipitation (DEMO4), 8‐20 precipitation (DEMO5), all-day precipitation (DEMO6), average air pressure (DEMO7), average air temperature (DEMO8), maximum air temperature (DEMO9), mean wind speed (DEMO10), maximum wind speed (DEMO11), small pan evaporation, big pan evaporation, minimum relative humidity, maximum land surface temperature, minimum land surface temperature, maximum air pressure, minimum air pressure, wind speed direction, and minimum air temperature were collected from the China Meteorological Data Sharing Service System. Data on gross domestic product (GDP), density of the population, and digital elevation model (DEM) were obtained from the website of the National Earth System Science Data Center, National Science & Technology Infrastructure of China. The data on the NDVI and the enhanced vegetation index (EVI) were obtained from the Geospatial Data Cloud. Approximately 19 bioclimatic factors including temperature seasonality (bio_4), precipitation of the wettest month (bio_13), and precipitation of the wettest quarter (bio_16) were collected from the World Climate Database. The data on density of cattle and density of sheep were collected from the Food and Agriculture Organization.

Since 2012, a total of 14 counties in Zhejiang Province, including Daishan, Dinghai, Linhai, Jiaojiang, Tiantai, Sanmeng, Ninghai, Anji, Chun’an, Jingning Autonomous, Pujiang, Yiwu, Shangyu, and Pingyang, conducted surveillance of tick species and density (Figure 1). The flag method was adopted to collect ticks and calculate the questing tick density. All ticks were identified to the species level, and the targeted species was *H. longicornis*. The tick surveillance program is implemented from March to October at a frequency of once a month. In terms of tick monitoring sites, 2 types of habitats including rural environments and scenic landscapes should be selected. For the selected village, at least 1 farmland (including a tea garden and other industrial crop fields), barren slope grassland, or woodland habitat around the village was selected to conduct the monitoring program. For the selected scenic area, including urban parks, country parks, forest parks, deserts, grasslands, and other man-made landscapes, at least one of the monitoring sites should be selected to carry out questing tick monitoring. Tick density was calculated based on the number of ticks captured per hour of each flag. Generally, each flag (90 cm long and 60 cm wide) must be dragged (waved) no less than 500 m for no less than 30 minutes including the time needed for the removal of the collected ticks from the flag.

### Data Processing

**Figure 2.**
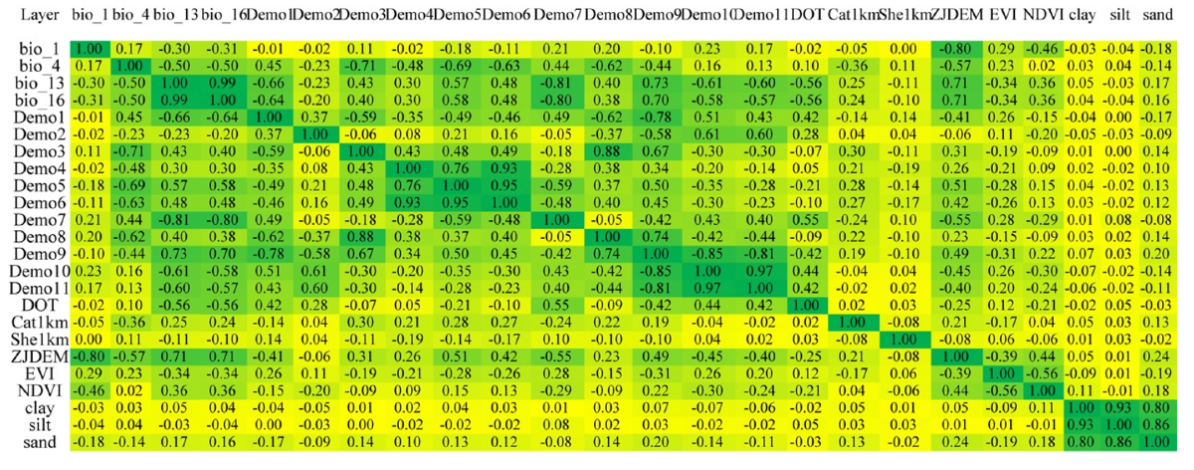
The result of cross-correlation analysis. A total of 24 factors including environmental factors, meteorological factors, and tick density were analyzed. The color transitioned from yellow to green representing the corresponding increase in |*r*| from 0 to 1. The factors that were highly correlated with others (Pearson |*r*|>0.8) were removed.

The meteorological data with several missing values were first filled with the *missForest* package in R software (version 4.0.2; The R Foundation). Then, the layers of meteorological factors from 2011 to 2019 were generated by a type of interpolation method called partial thin plate smoothing splines in the ANUSPLIN system and the kriging interpolation method in QGIS (version 3.14; QGIS Development Team). The layer for density of ticks (DOT) was generated by kernel density estimation with QGIS. In Figure 2, factors exhibiting a correlation coefficient with an absolute value exceeding 0.8 (Pearson |*r*|>0.8) are deemed to possess a strong correlation [23,24]. When faced with such strongly correlated factors, we proceed by retaining those factors that are likely to hold practical significance for SFTS, as determined through a thorough literature review and expert consultation. For factors whose relevance is less clear, we opt to initially include them within our model and subsequently use the jackknife method for further selection. This method, introduced by the esteemed statistician John Tukey, is widely recognized as a robust hypothesis-testing approach and shares conceptual parallels with the leave-one-out cross-validation technique. It facilitates an assessment of the individual impact that the removal of each factor has on the model’s estimated values and overall performance, as illustrated through a jackknife plot.

### Construction of the Maximum Entropy–Based Model

Three sets of factors were selected to build the models. The factors of set 1 model included bio_4, bio_13, bio_16, cattle 1km_ad, sheep 1km_ad, DEMO1, DEMO 2, DEMO 5, DEMO8, DOT, GDP, Sand, NDVI, and DEM; set 2 model included bio_4, bio_13, cattle 1km_ad, DEMO2, DEMO5, DEMO8, DOT, GDP, EVI, NDVI, and DEM; and set 3 model included bio_13, cattle1km_ad, DEMO1, DEMO2, DEMO8, DOT, GDP, EVI, NDVI, and DEM. All the layers of factors were rescaled with the same cell size and were extracted to the same dimensional geographic boundaries.

The maximum entropy (MaxEnt) model is an artificial intelligence model based on machine learning techniques, and the predictive performance is believed to almost be at the highest level for modeling ENM [25,26]. In this study, SFTS distribution from 2011 to 2019 in Zhejiang Province was regarded as a dependent factor; the remaining factors (described earlier) were independent factors.

Threshold independent receiver-operating characteristic (ROC) analysis was used to calibrate and verify the robustness of evaluation for MaxEnt. To evaluate the predictive precision of MaxEnt, an area under the receiver operating characteristic curve (AUC) was examined. The AUC value of 0.5 indicates whether the model predictions are better than random, a value of 0.5‐0.7 shows poor performance, 0.7‐0.9 forecasts reasonable performance, and >0.9 indicates high performance [27]. However, a previous study indicated that ROC and AUC did not provide information about the good performance of the model [28]. In this study, the partial ROC was calculated instead of the full area under the ROC curve. The model parameters were selected by the package *kuenm* in R; partial ROC and omission rates (ORs) are evaluated based on models created with training occurrences, whereas Akaike’s Information Corrected Criterion (AICc) values are calculated for models created with the full set of occurrences [27]. We had set regularization multipliers as 0.1, 0.2, 0.3, 0.4, 0.5, 0.6, 0.7, 0.8, 0.9, 1, 2, 3, 4, 5, 6, 8, and 10, and the feature classes as linear features, quadratic features, product features, threshold features, hinge features, and their permutation and combination. All candidate models with parameters reflecting all combinations of 17 regularization multiplier settings, 31 feature class combinations, and 3 distinct sets of environmental factors were evaluated. Model performance was evaluated based on statistical significance, OR, and the AICc. The function evaluates candidate models based on 3 distinct criteria: statistical significance (based on partial ROC analyses), prediction ability (ORs), and model fit and complexity (using AICc). Statistical significance was determined by bootstrap resampling 50% of the test data, and probability was assessed by a direct count of the percentage of bootstrap repeats with AUC ratios ≤1.0. We measured the model's predictive performance on the test data by using the OR, and we selected a threshold of *E* equal to 10%. We selected “OR_AICc” as the criterion for the final model selection. Models with an omission rate below a certain threshold were considered, and among these, those with the lowest AICc values and the smallest delta AICc values were chosen, as they met the omission rate criterion. The results indicated that the regularization multipliers were set to 0.4, and product features and quadratic features were selected. The maximum number of background points was set to 10,000, the maximum number of iterations was established at 500, and the convergence threshold was defined as 0.00001. We opted for the subsample (subsample method is more suitable for factors with nonnormal distributions) method for our analysis. Papers concerning ecologically suitable conservation areas have demonstrated that when the threshold rule is set to maximize test sensitivity and specificity, although there is a risk of overestimating the distribution, it minimizes the possibility of missing actual distribution areas [29,30]. For SFTS, it is crucial to ensure that we do not overlook the distribution in high-risk areas. Therefore, in this study, we have set the threshold rule to maximize test sensitivity and specificity. We split the remaining SFTS occurrences from 2011 to 2018 randomly into 50%‐50% subsets for model calibration and internal testing, respectively. The final models with no transfers were chosen for the prediction of the 2019 risk map, which will be compared with actual 2019 SFTS occurrence data.

In the evaluation process, a total of 3 sets of factors were designed to determine whether the factors chosen were appropriate. The factors of set 1 model included all the above factors; the set 2 model contained bio_4, bio_13, DEMO1, DEMO2, DEMO5, DEMO8, DOT, NDVI, SAND, density of sheep, density of cattle, and DEM; and the set 3 model included bio_13, DEMO8, DOT, NDVI, density of cattle, and DEM.

The number of all candidate models was 1581, and 1526 statistically significant models were simultaneously compared.

### Ethical Considerations

All methods were carried out in accordance with relevant guidelines and regulations. This study was reviewed and approved by the Ethics Committee of the Zhejiang Provincial Center for Disease Control and Prevention (2020‐021). The data used in this study were deidentified, ensuring that individual patients cannot be recognized. Prior to publication, the findings were thoroughly reviewed and approved by the ethics committee. No participants in the databases received any form of compensation.

## Results

### Descriptive Results

A total of 463 laboratory-confirmed SFTS cases were reported in Zhejiang Province between 2011 and 2019. Meanwhile, the epidemic center transferred from the northeast coast to the southeast coast. The ranges of bio_4, bio_13, bio_16, DEMO1, DEMO2, DEMO5, DEMO8, DOT, density of cattle, density of sheep, ZJDEM, NDVI, EVI, Sand, and GDP were 644.5‐921.3, 131.9‐351.9 mm, 339.7‐882.9 mm, 3.9‐5.5 hours, 72.3%‐83.4%, 573.5‐1115.8 mm, 16.9‐19.6 ℃, 0.2‐12.0/(hour*flag), 0‐90.3/km^2^, 0‐6775.7/km^2^, 0‐1918 m, 0.06‐1.0, 0.2‐1.0, 15.0‐84.0, and US $0‐$6825.7, respectively (Table 1).

**Table 1.** Ranges of different factors.

Factors	Description	Range
bio_4	Temperature seasonality	644.5‐921.3
bio_13	Precipitation of wettest month	131.9‐351.9 mm
bio_16	Precipitation of wettest quarter	339.7‐882.9 mm
DOT	Density of ticks	0.2‐12.0/(hour*flag)
DEMO1	Hours of sunshine	3.9‐5.5 hours
DEMO2	Average relative humidity	72.3%‐83.4%
DEMO3	Average land surface temperature	18.6‐22.5 ℃
DEMO4	20‐8 precipitation	587.1‐974.7 mm
DEMO5	8‐20 precipitation	573.5‐1115.8 mm
DEMO6	All-day precipitation	1134.0‐1943.7 mm
DEMO7	Average pressure	945.5‐1016.2 hPa
DEMO8	Average temperature	16.9‐19.6 ℃
DEMO9	Daily maximum temperature	19.8‐25.2 ℃
DEMO10	Mean wind speed	1.0‐6.4 m/s
DEMO11	Maximum wind speed	3.2‐10.4 m/s
GDP	Gross domestic product	US $0‐$6825.7
ZJDEM	Digital elevation model of Zhejiang	0‐1918 m
NDVI	Normalized difference vegetation index	0‐1
EVI	Enhanced vegetation index	0‐1
Cattle 1km_ad	Density of cattle	0‐90.3/km^2^
Sheep 1km_ad	Density of sheep	0‐6775.7/km^2^
Silt	Agrotype	8‐71
Sand	Agrotype	15‐84
Clay	Agrotype	7‐48

### Contributions of Different Factors

**Figure 3.**
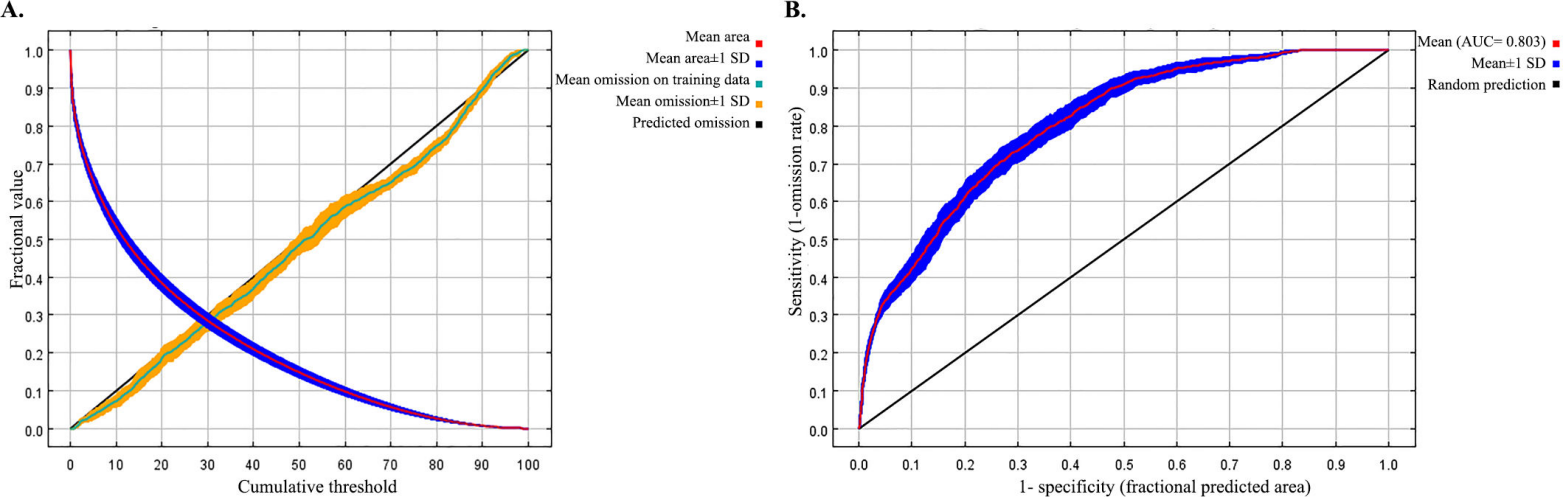
The receiver operating characteristic curve for the best model of set 3, which again averaged over the replicate runs, includes: (A) average omission and predicted area, and (B) average sensitivity vs 1- specificity for SFTS cases. The average test AUC for the replicate runs was 0.803 (SD 0.013). AUC: area under the receiver operating characteristic curve; SFTS: severe fever with thrombocytopenia syndrome.

The results of the 3 sets of models are summarized in Table 2. In the best set 1 model, the permutation importance of DOT, bio_13, DEMO8, density of cattle, DEM, NDVI, bio_4, DEMO1, DEMO2, DEMO5, density of sheep, SAND, bio_16, and GDP was 10.6%, 26.6%, 19.1%, 3.4%, 3.4%, 5.4%, 11%, 1.9%, 1.8%, 1%, 1.9%, 0.5%, 6.9%, and 5.7%, respectively. In the best set 2 model, the permutation importance of DOT, bio_13, DEMO8, density of cattle, DEM, NDVI, bio_4, DEMO1, and DEMO2 was 19.9%, 27.5%, 23.9%, 8%, 4.9%, 6.6%, 4.5%, 1.1%, and 3.6%, respectively. In the best set 3 model, the permutation importance of DOT, bio_13, DEMO8, density of cattle, DEM, and NDVI was 27.2%, 26.4%, 19.4%, 18.2%, 5%, and 3.8%, respectively. The estimates of relative contributions of DOT, bio_13, DEMO8, density of cattle, DEM, and NDVI were 23.2%, 22.1%, 15.6%, 15.4%, 11.9%, and 11.8% to the set 3 model. The ROC curve for the best model of set 3, which again averaged over the replicate runs is shown in Figure 3. The average test AUC for the replicate runs was 0.803, and the SD was 0.013. The jackknife sampling results for the regularized training gain across the models indicate that the omission of the DOT factor leads to the most significant decrease in gain. This suggests that DOT has the most substantial individual contribution that is not accounted for by the other factors (Multimedia Appendix 1).

**Table 2.** The permutation importance of factors in 3 sets of models.

Factors	Permutation importance (%)		
Set 1	Set 2	Set 3
DOT	10.6	19.9	27.2
bio_13	26.6	27.5	26.4
DEMO8	19.1	23.9	19.4
Cattle 1km_ad	3.4	8	18.2
ZJDEM	3.4	4.9	5
NDVI	5.4	6.6	3.8
bio_4	11	4.5	—^a^
DEMO1	1.9	1.1	—
DEMO2	1.8	3.6	—
DEMO5	1	—	—
Sheep 1km_ad	2.8	—	—
Sand	0.5	—	—
bio_16	6.9	—	—
GDP	5.7	—	—

a Not applicable.

### Risk Assessments of SFTS Occurrence

**Figure 4.**
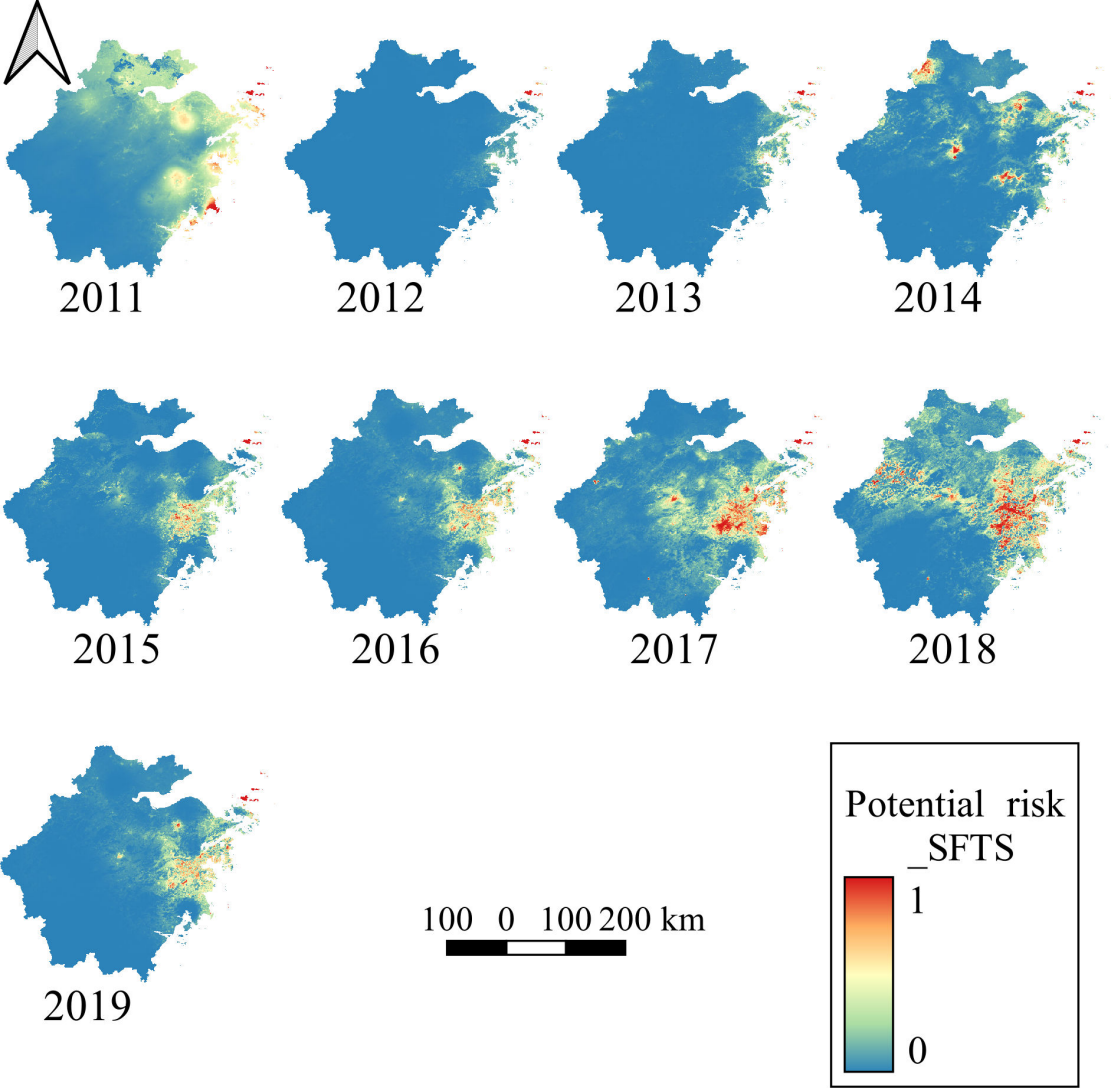
The maximum entropy prediction of SFTS risk areas. From 2011 to 2014, northeast Zhejiang Province had the highest risk, while risk in the east regions was similar to that of the northeast regions from 2015 to 2016. The east regions had the highest risk. SFTS: severe fever with thrombocytopenia syndrome.

Figure 4 shows the potential risk areas of SFTS occurrence. The results indicated that the northeast coast and central area of Zhejiang Province had the highest risk, and the affected areas expanded gradually. From 2011 to 2014, northeast Zhejiang Province had the highest risk, while the risk of east areas was similar to that of northeast areas from 2015 to 2016, and east areas had the highest risk since 2017.

**Figure 5.**
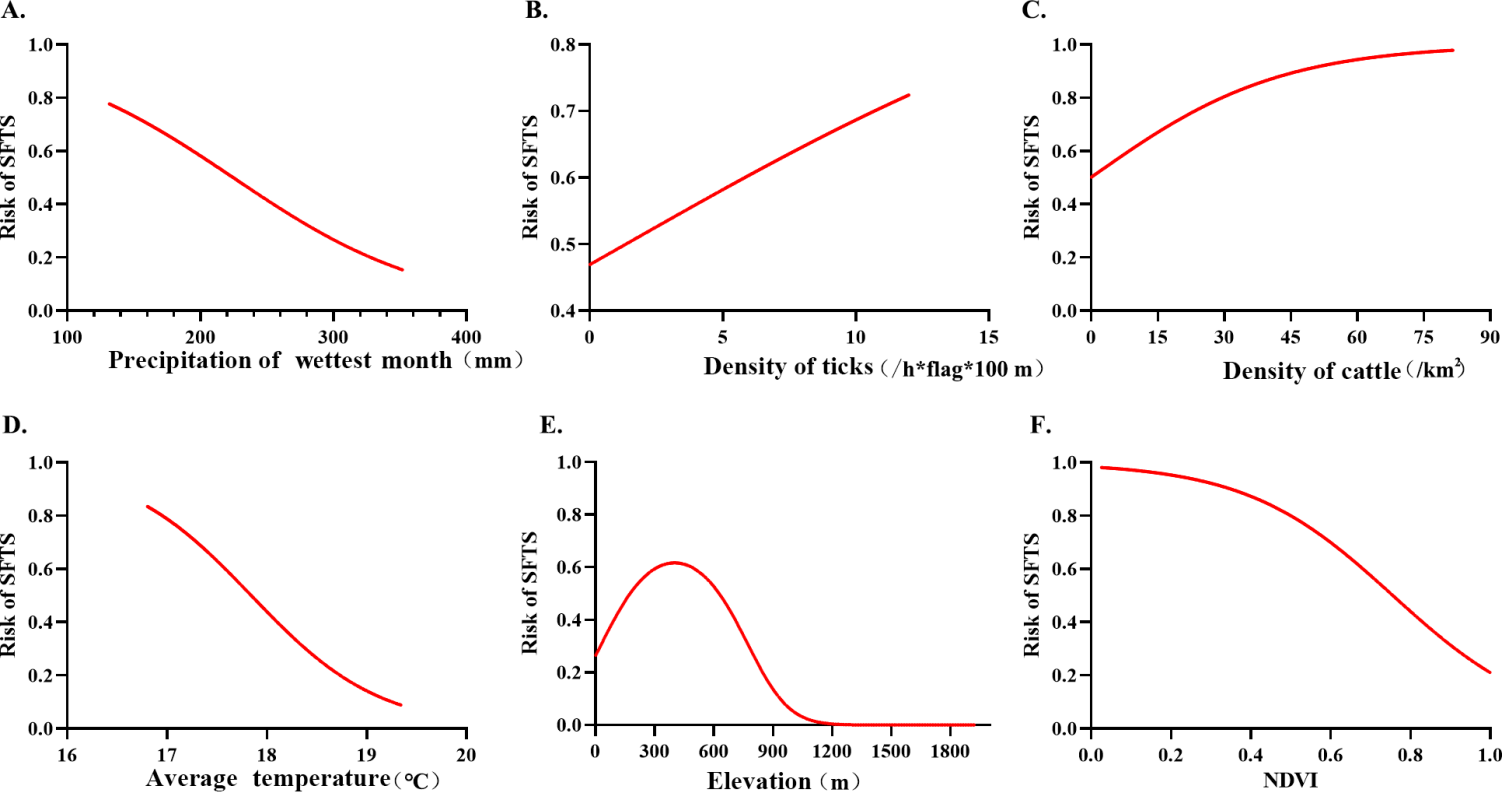
The predictive response curves for SFTS risk are based on associated factors: (A) precipitation of wettest month, (B) density of ticks, (C)density of cattle, (D)average temperature, (E) elevation, and (F) NDVI. The risk of SFTS occurrence increased with an increase in tick density and a decrease in precipitation in the wettest month, and it had a reverse V relationship with elevation. NDVI: normalized difference vegetation index; SFTS: severe fever with thrombocytopenia syndrome.

Figure 5 shows the response curves between independent and dependent factors. SFTS risk decreased with the increase of precipitation in the wettest month. When bio_13 ranged from 131.9 to 224.4 mm, the risk of SFTS occurrence was greater than 0.5, and when precipitation of the wettest month exceeded 224.4 mm, the risk probability was less than 0.5. When DOT exceeded 1.35/(hour*flag), the response curve indicated that the risk was greater than 0.5, and the risk of SFTS increased with the increase of tick density when cattle density ranged from 0.2 to 81.5/km^2^; the risk of SFTS increased with the increase of cattle density. When the average temperature ranged from 17.8 to 19.3 ℃, the risk of occurrence of SFTS cases was less than 0.5. For factor ZJDEM, risk probability presented an upward trend first, reached the peak at approximately 400 m, and then showed a downward trend. In addition, the risk probability of SFTS was greater than 0.5 when the altitude was between 112 and 408 m. The response curve between NDVI and SFTS risk is a left-skewed bell-shaped curve.

## Discussion

### Principal Findings

The results of the final model and the process of jackknife verified our conjecture on the critical factor of SFTS in Zhejiang Province; the factor DOT had the largest contribution to the model. Meanwhile, this study confirmed that SFTS incidence increased with the increase of tick density in Zhejiang Province.

More granular data can supplement public health sources to offer improved surveillance, and more research on influencing factors provides bases for developing measures for risk among humans [31,32]. Targeted intervention measures could be implemented according to the prediction results to prevent the occurrence of the epidemic. To predict the occurrence of SFTS, it is necessary to explore the factors associated with SFTS occurrence and the contribution rate of each factor and to establish an accurate prediction model.

MaxEnt is a robust species distribution model capable of predicting the potential distribution of a species based on its known occurrence records and environmental factors [33]. It is also adept at handling incomplete data sets and is particularly useful in scenarios where only presence data are available. Incorporating climate models, MaxEnt can forecast the potential effects of future climate change on species distributions, aiding managers in predicting and preparing for the challenges that climate change may present [33-35]. In this study, by integrating the MaxEnt model with Geographic Information System technology, we have been able to generate detailed distribution maps for SFTS. Furthermore, the MaxEnt model evaluates the impact of various environmental factors on SFTS, thereby identifying the key ecological factors that influence the risk distribution of SFTS. These maps illustrate the probability of SFTS occurrence, aiding managers and policy makers in understanding the geographical risk distribution of the disease, which is crucial for comprehending the disease’s risk control requirements and formulating protective measures. Accurate and anticipatory monitoring and early warning information for SFTS can assist managers in the efficient allocation of limited resources, such as focusing protective efforts on areas with a high-risk distribution of SFTS.

In this study, we first selected the 14 remaining factors as set 1 factors after processing the point sample and the cross-correlation analysis. Three sets of candidate models for evaluation and selection were generated. At the level of total SFTS cases from 2011 to 2018, the result of MaxEnt indicated that DOT, precipitation in the wettest month, average temperature, density of cattle, elevation, and NDVI were most important for the occurrence of SFTS cases. To further verify the suitability of these factors, we used these factors in 2019 as the future scenario and predicted the SFTS distribution in Zhejiang Province in 2019; we also verified the suitability of parameter setting of the set 3 model by the above factors. Set 3 model showed that density of cattle was also identified as a factor affecting the occurrence of SFTS. The density of cattle may indirectly reflect the prevalence of SFTSV in animal hosts, which in turn affects the risk of SFTS infection in humans. It was mentioned that the risk of SFTS increased with cattle density in the range of 0.2 to 81.5/km². This may be because higher cattle density may mean more tick hosts and thus may have increased tick and SFTSV transmission in the environment. Increased tick density directly increases human exposure to ticks and SFTSV, while bovine density may indirectly affect human SFTS risk by affecting SFTSV transmission in animal hosts. These findings have important implications for the development of targeted preventive measures and control strategies. Precipitation of the wettest month also significantly contributed to the occurrence of SFTS. In Zhejiang Province, we found a general trend, that is, the higher the value of precipitation of the wettest month, the lower the risk of SFTS. When the precipitation in the wettest month exceeded 224.4 mm, the risk probability of SFTS was less than 0.5. A previous study on a national level also showed that SFTS occurrence probability started to decrease after annual precipitation exceeding 1600 mm [19]. However, the months with the most precipitation are mostly from June to August, which is consistent with the peak of the temporal distribution of SFTS cases reported in Zhejiang Province [15]. On the contrary, a previous study reported that more precipitation increased the probability of tick infestation in central and western China [36]. The possible reasons might be that the annual rainfall of Zhejiang Province could exceed 1700 mm and the precipitation of the wettest month ranged from 300 to 400 mm in coastal areas, indicating that the precipitation in Zhejiang Province was significantly higher than that in areas of central and western China [37]. Further research should be conducted to explore the real relation between precipitation and SFTS occurrence.

In Zhejiang Province, the land area is predominantly composed of mountainous regions, which constitute 74.6% of the total area. Water bodies make up 5.1%, while the remaining 20.3% is comprised of flatlands. In this study, the response curve between NDVI and SFTS risk is a left-skewed bell-shaped curve. *Haemaphysalis longicornis* is a species often collected in pastures and meadows because of its tolerance to an arid environment; it tends to prefer an environment with a forest edge to grassland. SFTS was considered to occur in areas with farmers, farmland, tea gardens, and mountains [4]. However, according to the residential addresses obtained from the China Information System for Diseases Control and Prevention system, cases were located in village clusters or mountains with less vegetation.

In this study, the risk probability of elevation presented an upward trend first, reached the peak at about 400 m, and then became a downward trend. The results suggest that people living in hilly areas are the high-risk population for SFTS in Zhejiang Province. On one hand, ticks were widely distributed in Zhejiang Province, and farmers accounted for the majority of SFTS cases [38,39]. On the other hand, the development of an ecotourism industry and an increase in outdoor recreational chasers can increase human visits to tick-infested areas, thereby increasing tick-human contact rates.

Several previous studies indicated that the results of the model analysis vary across different provinces in China [19]. In Jiangsu Province, China, the distribution of SFTS natural foci was under the influence of multidimensional environmental factors, and the slope and maximum temperature of the warmest month were the key environmental risk factors [40]. This suggests that local adaptation is a very valuable tool for the prevention and control of emerging infectious diseases. The incidence of every infectious disease is influenced by natural and social factors. There are social, policy, and cost implications for effective tick control. Social, technical, and environmental factors must be considered to adapt appropriate strategies and measures. Daishan County, which had been mostly affected by SFTS, had considerable success in controlling ticks, and its experience may be instructive. The measures that significantly reduced the SFTS incidence mainly included clearance of breeding sites, killing of tick adults, and health education [41]. For other factors, we can only predict the distribution of high-risk areas according to the ENM; but for SFTS, the DOT can be controlled artificially by several economically feasible and effective methods.

The distribution of risk areas transferred from northeast areas to east areas. Several factors might have contributed to the results. First, comprehensive measures were conducted in northeast areas, and the SFTS incidence decreased in these areas. For example, Daishan County was a typical representative in northeast areas. Second, more attention was paid to SFTS in east areas. For example, in some counties of east areas, all patients with thrombocytopenia and leukocytopenia were asked to screen for SFTSV. Third, the changes in natural environmental and meteorological factors in northeast areas and east areas from 2011 to 2019 might also influence the SFTS incidence.

### Limitations

There are several limitations of our study. First, data on SFTS cases were collected from passive surveillance; underreporting may occur in some areas with poor detection capacity and availability of health facilities. Second, some wild animals might be hosts of SFTSV, and SFTS occurrence might be influenced by the species and density of some wild animals. However, no relevant province-wide surveillance study has been conducted in Zhejiang Province. More research should be conducted to clarify whether data on animals are correlated with SFTS occurrence in Zhejiang Province.

### Conclusions

Tick density, precipitation, and elevation were dominant influencing factors for SFTS, and comprehensive intervention measures should be adjusted according to these factors to reduce the SFTS incidence in Zhejiang Province.

## Supplementary material

10.2196/46070 Multimedia Appendix 1 Jackknife sampling results for the regularized training gain of best set 3 model.

## Abbreviations

AICc: Akaike's Information Corrected Criterion
AUC: area under the receiver operating characteristic curve
DEM: digital elevation model
DOT: density of ticks
ENM: ecological niche model
EVI: enhanced vegetation index
GDP: gross domestic product
MaxEnt: maximum entropy
NDVI: normalized difference vegetation index
OR: omission rate
ROC: receiver-operating characteristic
SFTS: severe fever with thrombocytopenia syndrome
SFTSV: severe fever with thrombocytopenia syndrome virus
