# Supplementary figures and images for: Factors Associated With the Spatial Distribution of Severe Fever With Thrombocytopenia Syndrome in Zhejiang Province, China: Risk Analysis Based on Maximum Entropy
Source: JMIR Public Health Surveill. 2024 Aug 2;10:e46070. doi: 10.2196/46070 (PMC11310739; doi:10.2196/46070)

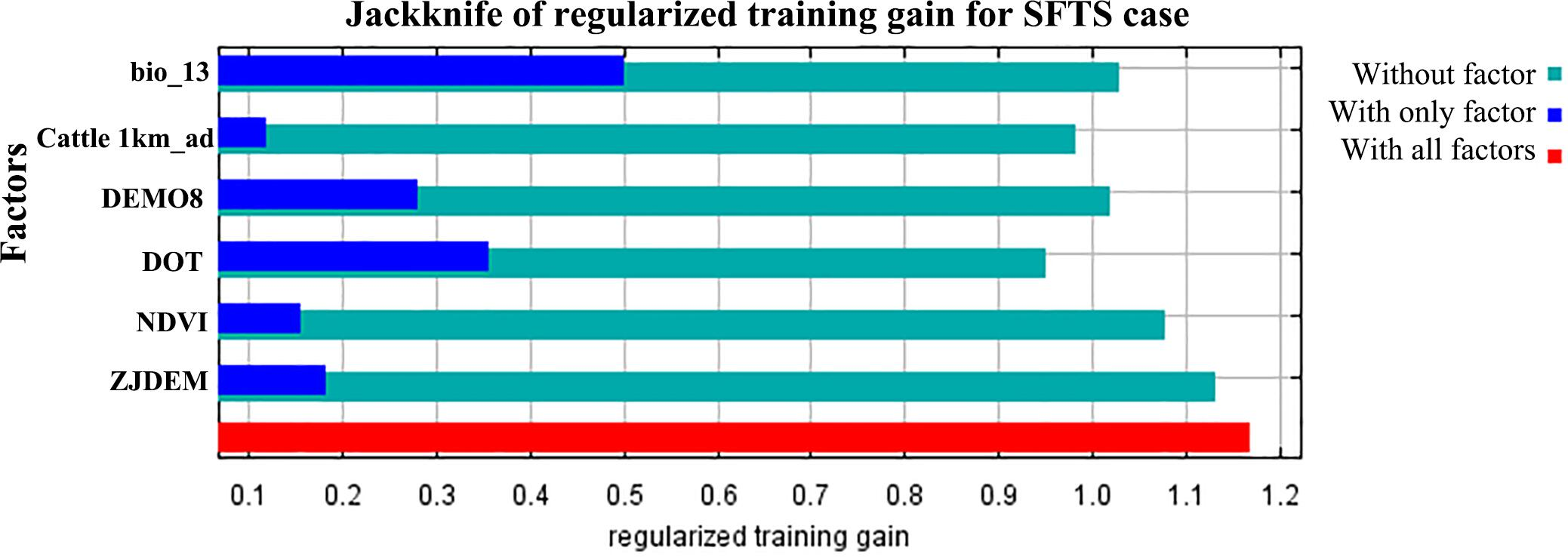

Supplement: Multimedia Appendix 1 [file publichealth-v10-e46070-s001.png]
